# Short Interpregnancy Intervals Among Women Experiencing Homelessness in Colorado

**DOI:** 10.1001/jamanetworkopen.2023.50242

**Published:** 2024-01-04

**Authors:** Rie Sakai-Bizmark, Nicholas J. Jackson, Frank Wu, Emily H. Marr, Hiraku Kumamaru, Dennys Estevez, Alison Gemmill, Jessica C. Moreno, Benjamin F. Henwood

**Affiliations:** 1 The Lundquist Institute for Biomedical Innovation at Harbor-UCLA (University of California, Los Angeles) Medical Center, Torrance; 2 Department of Pediatrics, Harbor-UCLA Medical Center and David Geffen School of Medicine at UCLA, Torrance; 3 Department of Medicine Statistics Core, David Geffen School of Medicine at UCLA, Los Angeles; 4 Department of Healthcare Quality Assessment, The University of Tokyo School of Medicine, Tokyo, Japan; 5 Department of Population, Family and Reproductive Health, Johns Hopkins Bloomberg School of Public Health, Baltimore, Maryland; 6 USC Suzanne Dworak-Peck School of Social Work, University of Southern California, Los Angeles

## Abstract

**Importance:**

Short interpregnancy intervals (SIPIs) are associated with increased risk of adverse maternal and neonatal outcomes. Disparities exist across socioeconomic status, but there is little information on SIPIs among women experiencing homelessness.

**Objective:**

To investigate (1) differences in rates and characteristics of SIPIs between women experiencing homelessness and domiciled women, (2) whether the association of homelessness with SIPIs differs across races and ethnicities, and (3) whether the association between SIPIs of less than 6 months (very short interpregnancy interval [VSIPIs]) and maternal and neonatal outcomes differs between participant groups.

**Design, Setting, and Participants:**

This cohort study used a Colorado statewide database linking the Colorado All Payer Claims Database, Homeless Management Information System, death records, and infant birth records. Participants included all women who gave birth between January 1, 2016, and December 31, 2021. Data were analyzed from September 1, 2022, to May 10, 2023.

**Exposures:**

Homelessness and race and ethnicity.

**Main Outcomes and Measures:**

The primary outcome consisted of SIPI, a binary variable indicating whether the interval between delivery and conception of the subsequent pregnancy was shorter than 18 months. The association of VSIPI with maternal and neonatal outcomes was also tested.

**Results:**

A total of 77 494 women (mean [SD] age, 30.7 [5.3] years) were included in the analyses, of whom 636 (0.8%) were women experiencing homelessness. The mean (SD) age was 29.5 (5.4) years for women experiencing homelessness and 30.7 (5.3) years for domiciled women. In terms of race and ethnicity, 39.3% were Hispanic, 7.3% were non-Hispanic Black, and 48.4% were non-Hispanic White. Associations between homelessness and higher odds of SIPI (adjusted odds ratio [AOR], 1.23 [95% CI, 1.04-1.46]) were found. Smaller associations between homelessness and SIPI were found among non-Hispanic Black (AOR, 0.59 [95% CI, 0.37-0.96]) and non-Hispanic White (AOR, 0.57 [95% CI, 0.39-0.84]) women compared with Hispanic women. A greater association of VSIPI with emergency department visits and low birth weight was found among women experiencing homelessness compared with domiciled women, although no significant differences were detected.

**Conclusions and Relevance:**

In this cohort study of women who gave birth from 2016 to 2021, an association between homelessness and higher odds of SIPIs was found. These findings highlight the importance of conception management among women experiencing homelessness. Racial and ethnic disparities should be considered when designing interventions.

## Key Points

**Question:**

Are the rates and characteristics of short interpregnancy intervals (SIPIs) different among women experiencing homelessness compared with domiciled women?

**Findings:**

In this cohort study of 77 494 participants from a statewide Colorado database, homelessness was associated with higher odds of SIPIs.

**Meaning:**

These findings highlight the importance of preventing SIPIs among women experiencing homelessness.

## Teaser

This cohort study examines differences in rates and characteristics of short interpregnancy intervals between women experiencing homelessness and domiciled women, including differences by race and ethnicity and by maternal and neonatal outcomes.

## Introduction

Short interpregnancy interval (SIPI) is defined as an interval between delivery and conception of the subsequent pregnancy shorter than 18 months. In the US, an estimated 29% to 35% of mothers have had an interpregnancy interval that is categorized as an SIPI.^1,2,3^ An SIPI is associated with adverse maternal and neonatal outcomes, such as preterm birth,^4,5,6,7,8^ infant death,^6,7,8^ and maternal mortality.^9,10^ Births following SIPIs were more likely to be described as mistimed or unwanted births, suggesting potential for reduction in SIPI births through contraceptive management and counseling.^2,11,12^ Gemmill and Lindberg^2^ estimated a 10% decrease in SIPI births by preventing unintended pregnancies.

Disparities in SIPI have been demonstrated across socioeconomic status (SES) and race and ethnicity.^3,11,13,14,15,16^ However, to our knowledge, there has been no attempt to evaluate SIPI among women experiencing homelessness who are disproportionately from racial and ethnic minority groups.^17^ In addition, homelessness is known to increase the risk of pregnancy complications and adverse maternal and neonatal outcomes, such as preterm delivery and neonatal intensive care unit admission.^18,19,20,21,22,23^ Infancy (younger than 1 year) is the age at which a person is most likely to spend a night in a shelter in the US.^24^ As infants are typically accompanied by their mothers, this statistic illustrates that women in the postpartum period are also at high risk of homelessness. Pregnancy can increase the risk of homelessness, as a new child is not welcome in some living situations.^25^ A complex array of challenges faced by women experiencing homelessness, including limited access to health care, sexual coercion and violence, unstable living conditions, economic stress, and a lack of social support, can lead to reduced control over family planning decisions and an increased risk of SIPI.^25,26,27^ The objectives of this study are to examine differences in rates and characteristics of SIPI between women experiencing homelessness and domiciled women, whether the association of homelessness with SIPI differs across race and ethnicity, and whether the association between interpregnancy intervals less than 6 months (very short interpregnancy intervals [VSIPIs]) and maternal and neonatal outcomes differs between women experiencing homelessness and domiciled women.

## Methods

This cohort study was reviewed and approved by the institutional review board at the Lundquist Institute for Biomedical Innovation at Harbor-UCLA Medical Center. Informed consent was waived by the institutional review board due to use of public health records and deindentified data. Data were analyzed from September 1, 2022, to May 10, 2023. This study followed the Strengthening and Reporting of Observational Studies in Epidemiology (STROBE) reporting guideline.

### Database

The database included the following linked datasets from January 1, 2016, to December 31, 2021, from the state of Colorado: (1) the All Payer Claims Database (CO APCD), which is a state-legislated database managed by the Center for Improving Value in Health Care^28^ and containing 100% of Medicaid claims and claims from 40 commercial health insurance plans (70% of the commercial market coverage); (2) Homeless Management Information System,^29^ a local information technology system used to collect client-level data and a requirement for municipalities to receive federal funding for homeless services; (3) death records; and (4) infant birth records. Death and birth records were obtained from the Vital Statistics Program, Colorado Department of Public Health and Environment.^30^ Notably, the CO APCD excludes records for individuals without insurance. Further details are provided in eTable 1 in Supplement 1.

### Identification of Patients

Using birth records, we identified infants born between 2016 and 2021. Using linked data, we identified the mothers of those infants. We only included records for women who had a previous pregnancy resulting in a live birth. Records from women whose previous pregnancy resulted in termination were excluded from the analyses, as the World Health Organization’s recommendation for interpregnancy interval following a loss differs from the recommended interval following a live birth.^31^ Other exclusion criteria are provided in the eMethods in Supplement 1. A total of 180 436 individuals (666 women experiencing homelessness and 179 770 domiciled women) were excluded from the analyses by these criteria.

### Measurements

Details of outcomes and adjustment variables are provided in eTable 2 in Supplement 1. The primary exposure variables were homelessness and race and ethnicity. We defined women experiencing homelessness as women who used shelter services at least once during the study period. Domiciled women, defined as women who never used shelter services, formed the comparison group. People who used shelters could have been domiciled at other times during the study period. However, using shelter services indicates the individuals faced housing insecurity and therefore belonged to the high-risk group that needed care. Race and ethnicity were identified from mother’s race and mother’s ethnicity as reported on infant birth certificates. Race and ethnicity categories included Hispanic, non-Hispanic Black, non-Hispanic White, and other (including American Indian or Alaskan Native, Asian, Native Hawaiian or Other Pacific Islander, and unspecified race or ethnicity).

The primary outcome of interest was a binary variable indicating whether the interpregnancy interval of the most recent delivery that occurred during the study period was shorter than 18 months. Interpregnancy interval was calculated as the length of time between the month of the previous live birth and the month of conception of the most recent pregnancy, which was calculated from the month of the most recent delivery and the gestational age (in weeks) listed in the infant’s birth record.

Based on widely known evidence that interpregnancy intervals less than 6 months (VSIPI) may be subject to different confounding factors than interpregnancy intervals between 6 and 18 months (SIPI),^15,31,32,33,34^ we evaluated the differences in the association of VSIPIs with maternal and neonatal outcomes between women experiencing homelessness and the comparison group of domiciled women. Specifically, the main factor affecting the association between VSIPIs and health outcomes may be physiological recovery from the prior pregnancy and delivery, whereas confounding factors such as health behaviors and access to health care resources may have a greater influence on the association between longer SIPIs and health outcomes. This perspective aligns with well-established physiological principles. Moreover, study designs that control for unobserved confounding, such as within-woman designs, show persistent associations between extremely short interpregnancy intervals and health outcomes, suggesting a true causal relationship.^4,32^ Therefore, we believe this approach is a reasonable strategy to comprehensively explore the implications of varying interpregnancy intervals on maternal and neonatal well-being. Adjustment variables were selected based on previous studies^2,6,10,13,15^ and data availability and collected from the birth record of the subsequent pregnancy (not the index pregnancy).

### Statistical Analysis

The distribution of interpregnancy intervals divided into 2-month increments was calculated. Multivariable logistic regression models were used to assess the association of homelessness with SIPI. We used marginal standardization^33^ to calculate risk-adjusted rates for SIPI stratified by homeless and domiciled status. All regression models were adjusted for the 4 adjustment variables listed in eTable 2 in Supplement 1 with a year fixed effect. Variance inflation factors were used to assess multicollinearity between adjustment variables, and values of 10 or higher were considered concerning. After accounting for adjustment variable associations, we examined whether homelessness and race and ethnicity were associated with differences in SIPI. To do this, we performed likelihood ratio tests to compare the 5 models listed in eTable 3 in Supplement 1. The interaction term between homelessness and race and ethnicity was added to the model to evaluate the potential moderating effect of race and ethnicity on the association of homelessness with SIPI. In this model, the adjusted odds ratio (AOR) for the interaction terms represents the difference in this association across various racial and ethnic groups. Multivariable logistic regression models with an interaction term between homelessness and VSIPI were used to assess differences in the association of VSIPI with the maternal and neonatal outcomes listed in eTable 2 in Supplement 1 between women experiencing homelessness and domiciled women.

A 2-sided *P* < .05 was considered statistically significant. We chose not to apply a multiple testing adjustment because our study aimed to comprehensively explore a wide range of associations among SIPI, homelessness, and maternal and neonatal outcomes. Implementing stringent adjustments increases the risk of type II errors, which could cause us to erroneously overlook genuine associations crucial for understanding the complex dynamics within this vulnerable population. Additionally, given the exploratory nature of our research, we prioritized capturing a comprehensive picture of these associations, acknowledging that future confirmatory studies could delve deeper into specific findings identified in this broad exploration. We used SAS, version 9.4. (SAS Institute, Inc), for the analyses, with the exception of estimating the adjusted rate, for which we used Stata, version 14 (StataCorp LLC).

We performed several sensitivity analyses. First, we defined women experiencing homelessness as women who used shelter services only before the more recent pregnancy. We excluded women who only used shelter services during the pregnancy or postpartum period from the analyses. Second, we used different cutoff values to define SIPI to test whether our definition affects our findings. We defined SIPI as an interpregnancy interval less than 3 months, less than 6 months, and less than 12 months. Third, to address outliers, we excluded interpregnancy intervals less than 3 months and greater than or equal to 60 months.

## Results

The final cohort consisted of 77 494 women with live births (mean [SD] age, 30.7 [5.3] years), of whom 636 were experiencing homelessness and 76 858 were domiciled women. In terms of race and ethnicity, 30 472 women (39.3%) were Hispanic, 5623 (7.3%) were non-Hispanic Black, 37 475 (48.4%) were non-Hispanic White, and 3924 (5.1%) were of other race or ethnicity. Detailed information on the records excluded based on the inclusion and exclusion criteria are shown in eTable 4 in Supplement 1. Due to the low number of missingness in the adjustment variables (ie, less than 0.5%), we used a complete case analysis. Compared with domiciled women, women experiencing homelessness were more likely to be younger (mean [SD] age, 29.5 [5.4] compared with 30.7 [5.3]), less likely to be non-Hispanic White (215 [33.8%] compared with 37 260 [48.5%]), more likely to be non-Hispanic Black (111 [17.5%] compared with 5512 [7.2%]) or Hispanic (286 [45.0%] compared with 30 186 [39.3%]), more likely to have public insurance (617 [97.0%] compared with 57 784 [75.2%]), less likely to be married (316 [49.7%] compared with 55 390 [72.1%]), and less likely to have a higher educational level (exact number for women experiencing homelessness is masked due to small size [<4%] compared with 19 464 [25.3%] for a bachelor’s degree or higher) (Table 1).

**Table 1.** Baseline Characteristics of Study Participants

Characteristic	Participant group^a^		*P* value
	Women experiencing homelessness (n = 636)	Domiciled women (n = 76 858)	
Individual			
Age, mean (SD), y	29.5 (5.4)	30.7 (5.3)	<.001
Age group, y			
<27	201 (31.6)	18 486 (24.1)	<.001
27-31	224 (35.2)	24 633 (32.1)	<.001
32-35	114 (17.9)	18 399 (23.9)	.09
>35	97 (15.3)	15 340 (20.0)	.003
Race and ethnicity			
Hispanic	286 (45.0)	30 186 (39.3)	<.001
Non-Hispanic Black	111 (17.5)	5512 (7.2)	<.001
Non-Hispanic White	215 (33.8)	37 260 (48.5)	.003
Other^b^	24 (3.8)	3900 (5.1)	.14
Social factors			
Insurance type			
Private	19 (3.0)	19 074 (24.8)	<.001
Public	617 (97.0)	57 784 (75.2)	<.001
Marriage			
Married	316 (49.7)	55 390 (72.1)	<.001
Never married	271 (42.6)	17 982 (23.4)	<.001
Widowed, separated, divorced	49 (7.7)	3486 (4.5)	<.001
Mother’s educational level			
≤8th Grade	≤10^c^	2953 (3.8)	<.001
9th-12th Grade	161 (25.3)	8828 (11.5)	<.001
High school graduate or GED completed	230 (36.2)	20 635 (26.8)	<.001
College credit but no degree	163 (25.6)	18 251 (23.7)	.27
Associate degree	53 (8.3)	6727 (8.8)	.71
Bachelor’s degree	16 (2.5)	12 184 (15.9)	<.001
Master’s degree	≤10^c^	5796 (7.5)	<.001
Doctorate or professional degree	0	1484 (1.9)	<.001
Clinical factors			
Plurality, mean (SD)^d^	1.0 (0.1)	1.0 (0.1)	.83
Single	627 (98.6)	75 691 (98.5)	.83
Twins	≤10^c^	1159 (1.5)	.85
Triplets	NR^e^	≤10^c^	.80
More	0	NR^e^	NA
Gestational age, mean (SD), wk	38.1 (2.3)	38.5 (1.8)	<.001
Maternal Comorbidity Index, mean (SD)	9.1 (11.5)	6.0 (9.4)	<.001
Cesarean delivery	192 (30.2)	19 595 (25.5)	.007
Maternal smoking	162 (25.5)	6640 (8.6)	<.001
Adequacy of Prenatal Care Utilization Index			
Inadequate	38 (6.0)	2220 (2.9)	<.001
Intermediate	114 (17.9)	12 915 (16.8)	.45
Adequate	192 (30.2)	29 769 (38.7)	<.001
Adequate Plus	254 (39.9)	29 561 (38.5)	.45
Body mass index^f^			
Underweight	NR^e^	5014 (6.6)	.07
Healthy weight	176 (27.7)	28 106 (37.0)	<.001
Overweight	143 (22.5)	20 233 (26.6)	.03
Obesity	256 (40.3)	22 648 (29.8)	<.001
Birth order			
2	229 (36.0)	39 689 (51.6)	<.001
3	162 (25.5)	21 246 (27.6)	.22
4	117 (18.4)	9671 (12.6)	<.001
>4	128 (20.1)	6252 (8.1)	<.001
Neighborhood income quartile			
1st (Lowest)	NA	18 085 (23.5)	NA
2nd	NA	21 022 (27.4)	
3rd	NA	21 029 (27.4)	
4th (Highest)	NA	16 567 (21.6)	
Missing	NA	155 (0.2)	

Abbreviations: NA, not applicable; NR, not reported.

^a^ Unless otherwise indicated, data are expressed as No. (%) of participants. Percentages have been rounded and may not total 100. Owing to missing data, some categories may not total numbers in column headings.

^b^ Includes American Indian or Alaskan Native, Asian, Native Hawaiian or Other Pacific Islander, and unspecified race or ethnicity.

^c^ Categories with less than 10 participants have been masked to protect patient privacy.

^d^ Indicates the number of infants delivered in the pregnancy.

^e^ Value is not reported to prevent obtaining counts in neighboring cells.

^f^ Calculated as weight in kilograms divided by height in meters squared.

Women experiencing homelessness had higher rates of SIPI compared with domiciled women (221 [34.7%] vs 21 729 [27.5%]). Results from the likelihood ratio tests showed that homelessness, race and ethnicity, and interaction terms between homelessness and race and ethnicity were associated with differences in SIPI (eTable 3 in Supplement 1). No variance inflation factors exceeded 10, indicating no problematic multicollinearity (eTable 5 in Supplement 1). Interpregnancy intervals of 6 to 7 months and 22 to 23 months were most frequent among women experiencing homelessness, whereas intervals of 14 to 15 months were most frequent among domiciled women (Figure). We found an association between homelessness and higher odds of SIPI (AOR, 1.23 [95% CI 1.04-1.46]; risk adjusted rate, 32.50% [95% CI, 28.99%-36.02%] among women experiencing homelessness vs 28.29% [95% CI, 27.98%-28.60%] among domiciled women) (Table 2). These results are consistent across sensitivity analyses (eTable 6 in Supplement 1).

**Figure.**
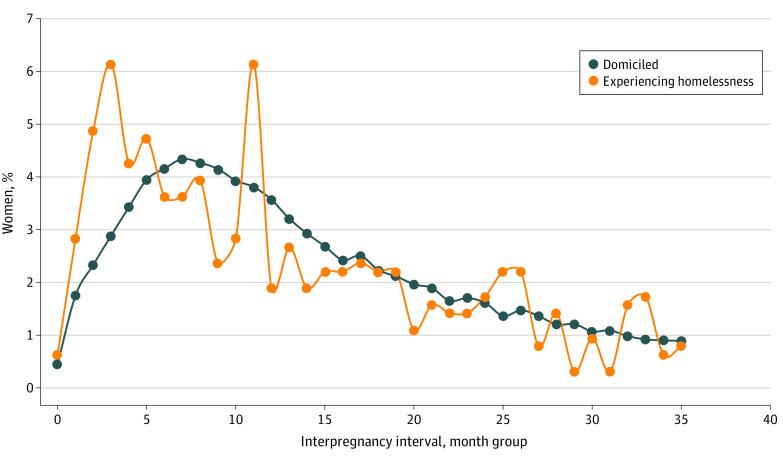
Distribution of Interpregnancy Intervals Stratified by Experiencing Homelessness and Domiciled Interpregnancy interval is defined as the number of months between delivery and conception of the subsequent pregnancy. Each dot depicts a 2-month group.

**Table 2.** The Regression Results to Evaluate the Association of Homelessness With Short Interpregnancy Interval

Participant group	AOR (95% CI)^a^	Adjusted rate (95% CI), %
Women experiencing homelessness	1.23 (1.04-1.46)	32.50 (28.99-36.02)
Domiciled women	1 [Reference]	28.29 (27.98-28.60)

Abbreviation: AOR, adjusted odds ratio.

^a^ Models were adjusted for age in quartile, race and ethnicity, insurance type, marital status, educational level, and birth order.

We found that the association between homelessness and SIPI was smaller among non-Hispanic Black (AOR, 0.59 [95% CI, 0.37-0.96]) and non-Hispanic White (AOR, 0.57 [95% CI, 0.39-0.84]) women compared with Hispanic women (Table 3 and eTable 7 in Supplement 1). We found a greater association of VSIPI with emergency department visits (AOR, 2.04 [95% CI, 0.87-4.32]) and low birth weight (AOR, 1.42 [95% CI, 0.31-6.57]) among women experiencing homelessness compared with domiciled women, although we did not detect any statistically significant differences (Table 4 and eTable 8 in Supplement 1).

**Table 3.** Difference in Association of Homelessness With Short Interpregnancy Interval Across Race and Ethnicity

Variable	AOR (95% CI)^a^
Housing status	
Women experiencing homelessness	1.63 (1.27-2.09)
Domiciled women	1 [Reference]
Race and ethnicity	
Hispanic	1 [Reference]
Non-Hispanic Black	1.54 (1.45-1.64)
Non-Hispanic White	1.42 (1.37-1.48)
Other^b^	1.34 (1.24-1.45)
Interaction term between housing status and race and ethnicity	
Hispanic and housing status	1 [Reference]
Non-Hispanic Black and housing status	0.57 (0.39-0.84)
Non-Hispanic White and housing status	0.59 (0.37-0.96)
Other race and housing status	1.12 (0.47-2.65)

Abbreviation: AOR, adjusted odds ratio.

^a^ Models were adjusted for age quartile, race and ethnicity, insurance type, marital status, educational level, maternal comorbidity, plurality, maternal smoking, adequacy of prenatal care utilization index, birth order, and body mass index.

^b^ Includes American Indian or Alaskan Native, Asian, Native Hawaiian or Other Pacific Islander, and unspecified race or ethnicity.

**Table 4.** Association of Interpregnancy Interval Shorter Than 6 Months With Maternal and Neonatal Outcomes

Outcome	AOR (95% CI)^a^
**Maternal **	
Readmission within 1 y after delivery	
SIPI status	
With SIPI	1.02 (0.84-1.24)
No SIPI	1 [Reference]
Housing status	
Women experiencing homelessness	1.63 (0.91-2.92)
Domiciled women	1 [Reference]
Interaction term between VSIPI and housing status	
Women experiencing homelessness and SIPI	0.80 (0.24-2.67)
Domiciled women and SIPI	1 [Reference]
ED visits within 1 y after delivery	
VSIPI status	
With SIPI	1.21 (1.11-1.32)
No SIPI	1 [Reference]
Housing status	
Women experiencing homelessness	2.11 (1.65-2.70)
Domiciled women	1 [Reference]
Interaction term between SIPI and housing status	
Women experiencing homelessness and SIPI	2.04 (0.87-4.32)
Domiciled women and SIPI	1 [Reference]
Readmission through ED within 1 y after delivery	
SIPI status	
With SIPI	0.87 (0.65-1.16)
No SIPI	1 [Reference]
Housing status	
Women experiencing homelessness	1.78 (0.97-3.27)
Domiciled women	1 [Reference]
Interaction term between SIPI and housing status	
Women experiencing homelessness and SIPI	0.62 (0.12-3.28)
Domiciled women and SIPI	1 [Reference]
**Neonatal **	
Preterm labor	
SIPI status	
With SIPI	1.47 (1.28-1.69)
No SIPI	1 [Reference]
Housing status	
Women experiencing homelessness	0.75 (0.45-1.26)
Domiciled women	1 [Reference]
Interaction term between SIPI and housing status	
Women experiencing homelessness and SIPI	0.64 (0.15-2.82)
Domiciled women and SIPI	1 [Reference]
Low birth weight	
SIPI status	
With SIPI	1.28 (1.13-1.46)
No SIPI	1 [Reference]
Housing status	
Women experiencing homelessness	0.85 (0.64-1.13)
Domiciled women	1 [Reference]
Interaction term between SIPI and housing status	
Women experiencing homelessness and SIPI	1.42 (0.31-6.57)
Domiciled women and SIPI	1 [Reference]
NICU admission	
SIPI status	
With SIPI	1.25 (1.10-1.41)
No SIPI	1 [Reference]
Housing status	
Women experiencing homelessness	1.00 (0.77-1.30)
Domiciled women	1 [Reference]
Interaction term between SIPI and housing status	
Women experiencing homelessness and SIPI	0.66 (0.19-2.29)
Domiciled women and SIPI	1 [Reference]

Abbreviations: AOR, adjusted odds ratio; ED, emergency department; NICU, neonatal intensive care unit; SIPI, short interpregnancy interval; VSIPI, very short interpregnancy interval.

^a^ Models were adjusted for age quartile, race and ethnicity, insurance type, marital status, educational level, maternal comorbidity, plurality, maternal smoking, adequacy of prenatal care utilization index, birth order, and body mass index. Length of stay during the delivery hospitalization was also included in the models to evaluate the association with maternal outcomes (ie, readmission within 1 year after delivery, ED visits within 1 year after delivery, and readmission through ED within 1 year after delivery).

## Discussion

Using a linked statewide Colorado database in this cohort study, we found that a significantly higher risk of SIPI among women experiencing homelessness. In addition, we found a smaller association of homelessness with SIPI among non-Hispanic White and non-Hispanic Black women compared with Hispanic women.

We found a higher rate of SIPI among women experiencing homelessness compared with domiciled women (34.7% vs 28.3%). This result is consistent with existing evidence demonstrating an association between low SES and SIPI.^13,14,15^ Compared with women with higher SES, women with low SES are less likely to use contraception and more likely to experience contraceptive failure, which increases their risk of unintended pregnancy.^34,35^ Unintended pregnancy, in turn, increases their risk of SIPI.^36^ Dehlendorf et al^35^ suggested that disparities in contraceptive use are due to a combination of individual-level preferences and behaviors, health care system factors, and clinician factors. For example, women of lower SES have less information about contraception, less access to family planning services, and more negative interactions with health care practitioners. Women experiencing homelessness face similar barriers to family planning as other women with low SES. Additionally, they experience unique barriers such as prioritizing the search for shelter over taking care of their health, lack of secure storage for contraceptives, and an increased vulnerability to sexual violence and coercion.^36^ Gelberg et al^26^ found that one-third of the women experiencing homelessness surveyed in Los Angeles County, California, rarely or never used contraception, leading to significantly higher rates of unintended pregnancy among women experiencing homelessness compared with domiciled women (73% vs 49%). Furthermore, it has been reported that unhoused youths engage in survival sex at high rates,^37,38,39^ which is often associated with the nonuse of barrier protection.^40^

We found variations in associations of homelessness with SIPI across different racial and ethnic groups. Racial and ethnic disparities within women experiencing homelessness have been reported previously, such as higher rates of unmet medical needs and lower rates of condom use among non-Hispanic White female homeless youths compared with their racial and ethnic counterpart groups.^40,41^

### Clinical Implications

Pregnancies during homelessness have been shown to be associated with adverse maternal and neonatal outcomes.^18,19,20,21,22,23,42,43^ Thus, VSIPI adds an additional layer of risk to these already high-risk pregnancies. Women experiencing homelessness encounter many different barriers to the access of health care services, such as cost, long wait times, uncertainty of where to go, lack of transportation, and difficulty remembering appointments.^44^ However, women experiencing homelessness are likely to deliver in a hospital, making hospital admissions an opportunity to provide counseling. Another optimal window to provide counseling may be around 6 to 7 months after a previous pregnancy, as that is when the highest rate of conception was observed among women experiencing homelessness in this study. To improve the comprehensive well-being of women experiencing homelessness, support from multisectoral care teams can play an important role. These teams should incorporate not only medical professionals, but also peer support staff and community-based programs.^45,46,47^

### Research Implications

Interventions to provide contraceptive services and clinical counseling aimed at increasing interpregnancy intervals, as well as resources aimed at reducing sexual exploitation, may be effective in reducing SIPI among women experiencing homelessness. These health care and family planning services should be tailored specifically to the needs and unique circumstances of women experiencing homelessness while also accounting for racial and ethnic disparities. The implications of our findings extend beyond clinical considerations to policy considerations. Policies that address this issue would benefit from a holistic approach that acknowledges homelessness among women as a convergence of diverse social determinants of health and behavioral factors. This recognition underscores that homelessness is not singularly a behavioral problem, but rather a complex interplay of multifaceted influences that necessitate tailored support and solutions. Ultimately, these policy implications underline the urgency of holistic support systems that bridge health care, housing, and social services to improve the reproductive outcomes of women experiencing homelessness and mitigate the disparities reported in the present study.

### Strengths and Limitations

To our knowledge, this is the first study to evaluate the association between homelessness and SIPI. Our innovative dataset, which included the linked CO APCD, Homeless Management Information System, death records, and infant birth records, allowed us to overcome several methodological limitations of existing literature focused on perinatal health issues among women experiencing homelessness. First, we were able to use a consistent definition of women experiencing homelessness across hospitals. Some hospital records and insurance claims data include variables indicating patients experiencing homelessness.^48,49^ However, the definition of patients experiencing homelessness is typically unknown and inconsistent across hospitals. Second, birth records provided important patient demographic information, such as the race and ethnicity of the mothers, which is often missing among patients with private insurance in the claims data.

This study also has some limitations. First, omitted variable bias cannot be ruled out because the study used observational data from secondary data sources. For example, we were not able to assess the effects of unmeasured factors, such as detailed SES, including occupation, pregnancy history (eg, maternal and neonatal complications), psychosocial factors (eg, mental health status and social support), health behaviors (eg, maternal nutrition and exercise habits), pregnancy intention, and access to health care resources. These factors may affect both SIPI and maternal and neonatal outcomes. Unfortunately, these variables were unavailable within our dataset, resulting in their omission from our analyses. Second, we defined women experiencing homelessness based on use of shelter services. As more than 68% of women experiencing homelessness in Colorado used some type of shelter services,^50^ we expected a small number of misclassifications. Additionally, if the women experiencing homelessness were misclassified as domiciled women, the bias would be toward the null.^51^ Furthermore, the number of women experiencing homelessness compared with domiciled women is very small, which leads to a small misclassification bias. Third, information on intervening pregnancy losses was based on the “date of last other pregnancy outcome (spontaneous or induced losses or ectopic pregnancies)” on the birth certificate. While poor data quality of this information has been documented,^52^ Conzuelo-Rodriguez et al^53^ reported that different methods to calculate interpregnancy intervals, considering live births, stillbirths, or miscarriages, yielded consistent results, especially to evaluate the association of VSIPI with preterm labor. Thus, this misclassification is less of a concern. Similarly, end dates of previous deliveries were obtained from the most recent birth certificates, rather than through a longitudinal linkage of birth records by mother. Although there is a possibility of measurement error, this method offers an advantage over the longitudinal linkage of statewide birth records, ensuring the inclusion of women relocating to other states between pregnancies. A study comparing this method with data from the National Survey of Family Growth reported similar interpregnancy interval distributions (30% and 29%, respectively, for intervals <18 months).^1^ Fourth, as we used the CO APCD, individuals who did not have any insurance were not included in the analyses. Moreover, since CO APCD encompasses 100% of Medicaid claims and 70% of commercial market claims, individuals with private insurance were more likely to be excluded from the analyses. To investigate the impact of these exclusions, we compared the demographic information available in the birth records of the study cohort with the records excluded from the study due to the absence of matching data in the claims records. Findings revealed that excluded records predominantly consist of individuals who are non-Hispanic White, are married, have a higher educational level, and have higher income levels (eTable 9 in Supplement 1). This exclusion introduces a potential bias toward the null, as individuals in higher SES groups were more likely to be excluded from the analyses. Fifth, as we only analyzed the data from Colorado and resources available for perinatal women experiencing homelessness vary by state, the results may not be generalizable to other parts of the country. Sixth, we did not examine maternal or infant mortality due to very small numbers. Multistate studies that include additional years of observation, and thus capture a larger number of maternal and infant mortality cases, will be of interest for future research.

## Conclusions

In this cohort study of women giving birth between 2016 and 2021, we found an association of homelessness with higher odds of SIPI and variations in associations of homelessness with SIPI across different racial and ethnic groups. Racial and ethnic disparities should be considered when designing interventions.

## Notes

Supplement 1. **eMethods.** Exclusion Criteria **eTable 1.** Detailed Explanation About the Data Source **eTable 2.** Data Source, Associated *ICD-10-CM*, and Explanation About the Variables **eTable 3.** Results From Likelihood Ratio Test (LRT) **eTable 4.** Analytic Cohort With Exclusion **eTable 5.** Variance Inflation Factors **eTable 6.** Association of Homelessness With Short Interpregnancy Interval: Sensitivity Analyses **eTable 7.** The Difference in Association of Homelessness With Short Interpregnancy Interval Across Race and Ethnicity **eTable 8.** Moderating Effect of Homelessness on the Association of Short Interpregnancy Interval With Maternal and Neonatal Outcomes **eTable 9.** Descriptive Statistics to Compare the Women Included in the Study and Women Excluded From the Study Due to the Absence of Matching Data in the Claims Records

Supplement 2.
Data Sharing Statement
