# Supplementary material for: Short Interpregnancy Intervals Among Women Experiencing Homelessness in Colorado
Source: JAMA Netw Open. 2024 Jan 4;7(1):e2350242. doi: 10.1001/jamanetworkopen.2023.50242 (PMC10767616; doi:10.1001/jamanetworkopen.2023.50242)
Supplement: Supplement 1. — eMethods. Exclusion Criteria eTable 1. Detailed Explanation About the Data Source eTable 2. Data Source, Associated ICD-10-CM, and Explanation About the Variables eTable 3. Results From Likelihood Ratio Test (LRT) eTable 4. Analytic Cohort With Exclusion eTable 5. Variance Inflation Factors eTable 6. Association of Homelessness With Short Interpregnancy Interval: Sensitivity Analyses eTable 7. The Difference in Association of Homelessness With Short Interpregnancy Interval Across Race and Ethnicity eTable 8. Moderating Effect of Homelessness on the Association of Short Interpregnancy Interval With Maternal and Neonatal Outcomes eTable 9. Descriptive Statistics to Compare the Women Included in the Study and Women Excluded From the Study Due to the Absence of Matching Data in the Claims Records [file jamanetwopen-e2350242-s001.pdf]

Supplementary Online Content

Sakai-Bizmark R, Jackson NJ, Wu F, et al. Short interpregnancy intervals among women experiencing homelessness in Colorado. *JAMA Netw Open*. 2024;6(1):e2350242. doi:10.1001/jamanetworkopen.2023.50242

eMethods. Exclusion Criteria
eTable 1. Detailed Explanation About the Data Source
eTable 2. Data Source, Associated *ICD-10-CM*, and Explanation About the Variables
eTable 3. Results From Likelihood Ratio Test (LRT)
eTable 4. Analytic Cohort With Exclusion
eTable 5. Variance Inflation Factors
eTable 6. Association of Homelessness With Short Interpregnancy Interval: Sensitivity Analyses
eTable 7. The Difference in Association of Homelessness with Short Interpregnancy Interval Across Race and Ethnicity
eTable 8. Moderating Effect of Homelessness on the Association of Short Interpregnancy Interval With Maternal and Neonatal Outcomes
eTable 9. Descriptive Statistics to Compare the Women Included in the Study and Women Excluded From the Study Due to the Absence of Matching Data in the Claims Records

This supplementary material has been provided by the authors to give readers additional information about their work.

© 2024 Sakai-Bizmark R et al. *JAMA Network Open*.

eMethods. Exclusion Criteria

1. We only included records for women whose previous pregnancy resulted in delivery. Records from women whose previous pregnancy resulted in termination were excluded from the analyses as the World Health Organization (WHO)'s recommendation for interpregnancy interval following a loss differs from the recommended interval following a live birth.[1]
2. For mothers who delivered multiple times during the study period, only the last delivery was included in the analyses to avoid serial correlations across our six-year study period, i.e., the within-person correlation across time.
3. Women whose delivery was their first delivery were excluded, as they do not have an interpregnancy interval from the previous delivery.
4. Women whose end date of prior pregnancy was missing from their records were excluded.
5. Women whose infants have a recorded gestational age at birth longer than the interval between deliveries were excluded, as either gestational age or the interval between deliveries is likely recorded incorrectly.
6. We excluded women whose records did not match any records in the HMIS database identifying them as women experiencing homelessness and also did not have a numeric Colorado zip code identifying them as domiciled women.
7. We excluded records missing any of the following individual information: i] race and ethnicity, ii] insurance type at delivery, iii] age, iv] gestational week, v] marital status, and vi] mother's education.

1. World Health Organization. *Report of a WHO technical consultation on birth spacing: Geneva, Switzerland 13-15 June 2005.* World Health Organization.; 2007 2007.

© 2024 Sakai-Bizmark R et al. *JAMA Network Open*.

eTable 1. Detailed Explanation About the Data Source

Process to Construct the Linked Database	
**Action**	**Details**
HMIS sent data for women between 18 years old and 47 years old to CO APCD	Data sent to CO APCD to aid with matching included name, birthdate, race and ethnicity, full SSN (last 4 digits of SSN, if full SSN was not available)<br><br>The following study variables were identified from the HMIS records:<br>1. HMIS project start date<br>2. HMIS project end date
CO APCD used ICD-9-CM and ICD-10-CM codes provided by the research team to identify women in the HMIS data who delivered babies. Each woman was assigned a study ID, which has been used across CO APCD and vital statistics.	The following study variables were identified from the CO APCD records:<br>1. Postpartum readmission<br>2. Postpartum readmission through the ED<br>3. Postpartum ED visits<br>4. Length of stay during the delivery hospitalization<br>5. Maternal comorbidity index score
CO APCD sent the study IDs to vital statistics and vital statistics identified infant birth and maternal death records with matching study IDs	The following study variables were identified from the birth records:<br>1. Interpregnancy interval (calculated as the length of time between the month of the previous live birth and the month of conception of the most recent pregnancy, which was calculated from the month of the most recent delivery and the gestational age [in weeks])<br>2. Mother's race<br>3. Mother's ethnicity<br>4. Mother's age<br>5. Insurance type<br>6. Marital status<br>7. Mother's education<br>8. Body mass index<br>9. Birth order<br>8. Preterm birth<br>9. Low birth weight<br>10. NICU admission<br>11. Maternal smoking<br>12. Maternal substance abuse<br>13. APNCU index
Vital statistic sent all records identified using the study IDs to the research team	The HMIS project start and end date were used by the study team to identify women who were homeless before, during, or after pregnancy

Abbreviations:
APNCU = Adequacy of prenatal care utilization
CO APCD = Colorado all payer claims database
ED = emergency department
HMIS = Homeless Management Information system
ICD-9-CM = International Classification of Diseases, Ninth Revision, Clinical Modification
ICD-10-CM = International Classification of Diseases, Tenth Revision, Clinical Modification
ID = identification
NICU = Neonatal Intensive Care Unit
SSN = social security number

© 2024 Sakai-Bizmark R et al. *JAMA Network Open*.

eTable 2. Data Source, Associated *ICD-10-CM*, and Explanation About the Variables

VARIABLE	DATA SOURCE	ICD-10-CM AND OTHER INFORMATION
**Outcome**		
Short interpregnancy interval	Infants' birth records	Interpregnancy interval was calculated as the length of time between the month of the previous live birth and the month of conception of the most recent pregnancy, which was calculated from the month of the most recent delivery and the gestational age (in weeks) listed in the infant's birth record.
**Exposure variables**		
Homelessness	Homeless Management Information System	We defined women experiencing homelessness as women who used shelter services at least one time during the study period.
Race and ethnicity	Infants' birth records	Women's race information was collected as mother's race from the infants' birth records because race information was not collected for patients with private insurance. Race and ethnicity were categorized as non-Hispanic White, non-Hispanic Black or African American, Hispanic, or other
**Maternal outcomes**		
Readmission within one year after the delivery	Infants' birth records, claims data	Readmission was identified in the claims data by calculating days between delivery discharge date and inpatient claims record within 365 days.
Emergency department (ED) visit within one year after the delivery	Infants' birth records, claims data	ED visit was identified in the claims data.
Readmission through ED within one year after the delivery	Infants' birth records, claims data	Readmission and ED visit was identified in the claims data by calculating days between delivery discharge date and ED claims record within 365 days.
**Neonatal outcomes**		
Preterm labor	Infants' birth records, claims data	O60, Z2A2, Z3A.30 - Z3.A36
Low birth weight	Infants' birth records	Infants' birth weights are recorded in their birth records
Newborn intensive care unit (NICU) admission	Infants' birth records	NICU admission is recorded in their birth records
**Adjustment variables included in all regression models**		
Age	Claims data	Age was included in the model in quartiles (<24 years, 24–28 years, 29–32 years, and >32 years)
Insurance type	Claims data	Public or private
Marital status	Infants' birth records	i] married, ii] never married, or iii] widowed, separated or divorced
Mother's education	Infants' birth records	i] ≤ 12th grade, ii] high school graduate or GED completed, and iii] possesses college credit. (More granular information for mother's education was available, (i.e., i] ≤8th grade, ii] 9th-12th grade, iii] high school graduate or GED completed, iv] college credit but no degree, v] Associate degree [e.g., AA, etc.], vi] Bachelor's degree, vii] Master's degree, viii] Doctorate or professional degree). However, we categorized the information into three groups due to a very small portion of women experiencing homelessness with education of ≤8th grade or higher than a Bachelor's degree.)
Maternal comorbidity	Claims data	A validated maternal comorbidity index score based on prenatal conditions
Plurality	Infants' birth records, claims data	Plurality is recorded in their birth records
**Adjustment variables included in the maternal and neonatal outcome regression models**		
Maternal smoking	Infants' birth records	Maternal Smoking is recorded in birth records
Substance abuse	Claims data	O99.31 - O99.33, F1.01, F1.11, F1.21, F1.31, F1.41, F1.51, F1.61, F1.71, F1.81, F1.91

© 2024 Sakai-Bizmark R et al. *JAMA Network Open*.

Birth order	Infants' birth records	Birth order was calculated from number of previous pregnancies alive, and number of previous pregnancies deceased.
Body mass index	Infants' birth records	Body mass index (BMI) was calculated from mother's height and pre-pregnancy weight. BMI was categorized into the following four groups: 1] Underweight (BMI < 18.5), 2] Healthy weight (18.5 to <25), 3] Overweight (25 to <30), and 4] Obesity (>=30)
**Adjustment variables included in the maternal and neonatal outcome regression models**		
Adequacy of prenatal care utilization index (APNCU)	Infants' birth records	Adequacy of prenatal care utilization (APNCU) index is calculated from gestational age at initiation of prenatal care and number of prenatal care visits. Based on these two independent and distinctive dimensions, APNCU index is categorized into the following four groups: 1] inadequate, 2] intermediate, 3] adequate and 4] adequate plus.
**Adjustment variables included only in the maternal outcome regression models**		
Delivery length of stay	Claims data	Length of stay is recorded in claims data.

© 2024 Sakai-Bizmark R et al. *JAMA Network Open*.

eTable 3. Results From Likelihood Ratio Test (LRT)

	LR Statistic[a]	DF[b]	p-value
Model 1: Only adjustment variables (Without homelessness or race or ethnicity)			
Model 2: Model 1 with homelessness	7.06	1	0.008
Model 3: Model 1 with race and ethnicity	395.65	3	<0.001
Model 4: Model 1 with homelessness, race and ethnicity	394.35	3	<0.001
Model 5: Model 1 with homelessness, race, ethnicity, and interaction term between homelessness and race and ethnicity	10.71	3	0.013

[a] The likelihood ratio test statistics

[b] Degrees of freedom

© 2024 Sakai-Bizmark R et al. *JAMA Network Open*.

eTable 4. Analytic Cohort With Exclusion

	Total	Women experiencing homelessness	Domiciled
	**No (%)**	**No (%)**	**No (%)**
**Delivery Hospitalizations**	257,930	1,302	256,628
Non-last delivery for women who had multiple deliveries during the study period, the analyses included only the last delivery.	57,059	392 (30.1)	56,667 (22.1)
No prior delivery	75,409	181 (13.9)	75,225 (29.3)
Prior delivery was termination	[b]	≤10 [a]	453 (0.2)
End date of prior delivery was missing	[b]	[b]	10,558 (4.1)
Records were excluded in which the records did not match with any records in the Colorado All Payers Claims Database	35,608	19 (1.5%)	35,589 (13.9)
Gestational week is longer than interval between deliveries	[b]	≤10 [a]	88 (0.03)
Records without all the following data at delivery hospitalization, 1] race/ethnicity, 2] insurance type during delivery, 3] age, 4] gestational week, 5] marital status, 6] mother's education	[b]	[b]	1,169 (0.5)
**Final Analytic Cohort**	77,494 (30.0)	636 (48.9)	76,858 (30.0)

[a] Cases ≤10 have been masked to protect patient privacy

[b] Case number has been masked to prevent obtaining counts in neighboring cells

© 2024 Sakai-Bizmark R et al. *JAMA Network Open*.

eTable 5. Variance Inflation Factors

VARIABLES	VARIANCE INFLATION
**SIPI[a]**	
SIPI	1.05
no SIPI	Reference
**Housing status**	
Experiencing homelessness	1.00
Domiciled	Reference
**Age group**	
Age <27	2.27
Age 27-31	1.97
Age 32-35	1.70
Age >35	Reference
**Race and ethnicity**	
Non-Hispanic White	5.53
Non-Hispanic Black	2.30
Hispanic	5.46
Other Race[b]	Reference
**Marital status**	
Married	4.84
Not Married	4.84
Widowed/Separated/Divorced	Reference
**Mothers' education**	
8th-High School graduate	2.06
Some College credit	1.59
College degree	Reference
**Plurality**	
Plurality 1	1.04
Plurality >= 2	Reference
**Insurance type**	
Private Insurance	1.44
Public Insurance	Reference
**Prenatal care**	
Prenatal: Inadequate	1.05
Prenatal: Intermediate	1.20
Prenatal: Adequate	1.22
Prenatal: Adequate Plus	Reference
**Comorbidity Score**	
Comorbidity Score	1.10
**Smoking**	
Smoking	1.31
No smoking	Reference
**Substance abuse**	
Substance abuse	1.29

© 2024 Sakai-Bizmark R et al. *JAMA Network Open*.

No substance abuse	Reference
**Birth Order**	
2nd birth	Reference
3rd birth	1.18
4th birth	1.21
5th birth or greater	1.25
**Body mass index**	
Underweight	1.15
Healthy weight	1.47
Overweight	1.38
Obesity	Reference

[a] Short interpregnancy interval

[b] The following races and ethnicities were included in the 'Other' category: Non-Hispanic American Indian or Alaskan Native, Non-Hispanic Asian, Non-Hispanic Native Hawaiian or Other Pacific Islander, and Non-Hispanic other (unspecified) race.

© 2024 Sakai-Bizmark R et al. *JAMA Network Open*.

eTable 6. Association of Homelessness With Short Interpregnancy Interval: Sensitivity Analyses

	aOR[a] (95% CI[b])	Adjusted rate (95% CI)
**Sensitivity analysis 1[c]**		
Women experiencing homelessness	1.15 (0.90-1.48)	31.11 (26.03-36.19)
Domiciled women	Reference	28.31 (28.00-28.62)
**Sensitivity analyses 2-1: IPI[d] < 3 months**		
Women experiencing homelessness	1.11 (0.62-1.98)	1.30 (0.70-2.04)
Domiciled women	Reference	1.18 (1.10-1.26)
**Sensitivity analyses 2-2: IPI[d] < 6 months**		
Women experiencing homelessness	1.32 (0.99-1.76)	5.09 (4.32-7.37)
Domiciled women	Reference	4.53 (4.38-4.68)
**Sensitivity analyses 2-3: IPI[d] < 12 months**		
Women experiencing homelessness	1.39 (1.15-1.68)	19.31 (16.51-22.11)
Domiciled women	Reference	14.88 (14.63-15.13)
**Sensitivity analyses 3: exclude IPI[d] less than three months and greater than 60 months**		
Women experiencing homelessness	1.24 (1.03-1.48)	40.91 (36.66-45.16)
Domiciled women	Reference	36.02 (35.63-36.40)

Models were adjusted for age in quartile, race and ethnicity, insurance type, marital status, education level, and birth order.
[a] Adjusted odds ratio
[b] Confidence interval
[c] We defined women experiencing homelessness as women who used shelter services only before the more recent pregnancy, which was identified based on the shelter service start date, year, and month of the more recent delivery, as well as the gestational age of the baby at birth (in weeks).
[d] Interpregnancy interval

© 2024 Sakai-Bizmark R et al. *JAMA Network Open*.

eTable 7. The Difference in Association of Homelessness With Short Interpregnancy Interval Across Race and Ethnicity

		Hispanic	Non-Hispanic White	Non-Hispanic Black	Other[a]
**Women experiencing homelessness**	aOR[b] (95% CI[c])	1.63 (1.27-2.09)	1.31 (0.98-1.77)	1.49 (0.99-2.23)	2.45 (1.07-5.57)
**Domiciled women**	aOR (95% CI)	Reference	1.42 (1.37-1.48)	1.54 (1.45-1.64)	1.34 (1.24-1.45)

Models were adjusted for age quartile, race, ethnicity, insurance type, marital status, education level, maternal comorbidity, plurality, maternal smoking, adequacy of prenatal care utilization index, parity and body mass index (BMI).

[a] The following races and ethnicities were included in the 'Other' category: Non-Hispanic American Indian or Alaskan Native, Non-Hispanic Asian, Non-Hispanic Native Hawaiian or Other Pacific Islander, and Non-Hispanic other (unspecified) race.

[b] Adjusted odds ratio

[c] Confidence interval

© 2024 Sakai-Bizmark R et al. *JAMA Network Open*.

eTable 8. Regression Results to Evaluate the Moderating Effect of Homelessness on the Association of Short Interpregnancy Interval with Maternal and Neonatal Outcomes

	**No SIPI[a]**	**SIPI**
	aOR[b] (95% CI[c])	aOR (95% CI)
**MATERNAL OUTCOMES**		
**Readmission within one year after delivery**		
Women experiencing homelessness	1.63 (0.91-2.92)	1.32 (0.47-3.76)
Domiciled Women	Reference	1.02 (0.84-1.24)
**ED visits within one year after the delivery**		
Women experiencing homelessness	2.11 (1.65-2.70)	5.21 (2.54-10.70)
Domiciled Women	Reference	1.21 (1.11-1.32)
**Readmission through ED within one year after the delivery**		
Women experiencing homelessness	1.78 (0.97-3.27)	0.97 (0.20-4.63)
Domiciled Women	Reference	0.87 (0.65-1.16)
**NEONATAL OUTCOMES**		
**Preterm labor**		
Women experiencing homelessness	0.75 (0.45-1.26)	0.71 (0.22-2.33)
Domiciled Women	Reference	1.48 (1.28-1.69)
**Low birth weight**		
Women experiencing homelessness	0.85 (0.64-1.13)	1.55 (0.41-5.86)
Domiciled Women	Reference	1.29 (1.13-1.46)
**NICU admission**		
Women experiencing homelessness	1.00 (0.77-1.31)	0.83 (0.24-2.81)
Domiciled Women	Reference	1.25 (1.10-1.42)

Models were adjusted for age in quartile, race/ethnicity, insurance type, marital status, education level, maternal comorbidity, plurality, maternal smoking, adequacy of prenatal care utilization index, birth order, and BMI. Length of stay during the delivery hospitalization was also included in the models to evaluate the association with maternal outcomes (i.e., readmission within one year after delivery, ED visits within one year after delivery, and readmission through ED within one year after delivery).

[a] Short interpregnancy interval

[b] Adjusted odds ratio

[c] Confidence interval

© 2024 Sakai-Bizmark R et al. *JAMA Network Open*.

eTable 9. Descriptive Statistics to Compare the Women Included in the Study and Women Excluded From the Study Due to the Absence of Matching Data in the Claims Records

	Study cohort	Individuals excluded from study due to incomplete linkages to APCD[a] data	P value
	77,494	35,608	
	**No. (%)**	**No. (%)**	
**INDIVIDUAL CHARACTERISTICS**			
**Housing Status**			
Women experiencing homelessness	636 (0.8)	19 (0.1)	<0.001
Domiciled women	76,858 (99.2)	35589 (99.9)	<0.001
**Race and Ethnicity**			
Hispanic	30,472 (39.3)	9227 (25.9)	<0.001
Non-Hispanic Black	5,623 (7.2)	1622 (4.6)	<0.001
Non-Hispanic White	37,475 (48.4)	22812 (64.0)	<0.001
Other[b]	3,924 (5.1)	1891 (5.3)	0.079
Unknown	0 (0)	56 (0.2)	<0.001
**SOCIAL FACTORS**			
**Marriage**			
Married	55,706 (71.9)	31,027 (87.1)	<0.001
Never Married	18,253 (23.5)	3,959 (11.1)	<0.001
Widowed, Separated, Divorced	3,535 (4.6)	622 (1.8)	<0.001
**Mother's Education**			
1] ≤8th grade	2,962 (3.8)	653 (1.8)	<0.001
2] 9th-12th grade	8,989 (11.6)	1,803 (5.1)	<0.001
3] high school graduate or GED completed	20,865 (26.9)	5,681 (15.9)	<0.001
4] college credit but no degree	18,414 (23.8)	7,188 (20.2)	0.022
5] Associate degree	6,780 (8.8)	3,264 (9.2)	<0.001
6] Bachelor's degree	12,200 (15.7)	10,248 (28.8)	<0.001
7] Master's degree	5,800 (7.5)	4,806 (13.5)	<0.001
8] Doctorate or professional degree.	1,484 (1.9)	1,607 (4.5)	<0.001
Unknown/Missing	0 (0)	358 (1.0)	<0.001
**CLINICAL FACTORS**			
Plurality (Mean and SD)	1.02 (0.1)	1.00 (0.1)	<0.001
Single	76,318 (98.5)	35,284 (99.1)	<0.001
Twins	1,168 (1.5)	323 (0.9)	<0.001
Triplet	≤10[c] ([d])	≤10[c] ([d])	0.188
More	[d] ([d])	[d] ([d])	NA
Gestational week (Mean and SD)	38.52 (1.8)	38.53 (1.8)	0.008
Cesarean Section	19,787 (25.5)	9,903 (27.8)	<0.001
Maternal Smoking	6,802 (8.8)	1,944 (5.5)	<0.001
**Adequacy of Prenatal Care Utilization Index**			
Inadequate	2,258 (2.9)	838 (2.4)	<0.001
Intermediate	13,029 (16.8)	5,383 (15.1)	<0.001
Adequate	29,961 (38.7)	14,870 (41.8)	<0.001

© 2024 Sakai-Bizmark R et al. *JAMA Network Open*.

Adequate Plus	29,815 (38.5)	13,358 (37.5)	0.002
Missing	2,431 (3.1)	1,159 (3.2)	0.294
**Body Mass Index (BMI)**			
Under	5,067 (6.5)	2,175 (6.1)	0.006
Healthy	28,106 (36.5)	15,294 (42.9)	<0.001
Over	20,233 (26.3)	9,245 (26.0)	0.241
Obesity	22,648 (29.6)	8,468 (23.8)	<0.001
Missing	865 (1.1)	426 (1.2)	0.246
**Birth order**			
2	39,918 (51.5)	21,649 (60.8)	<0.001
3	21,408 (27.6)	8,848 (24.9)	<0.001
4	9,671 (12.7)	3,345 (9.4)	<0.001
>4	6,252 (8.2)	1,765 (5.0)	<0.001
**NEIGHBORHOOD FACTORS**			
**Income Quartile**			
1st quartile (lowest)	18,312 (23.6)	6,004 (16.9)	<0.001
2nd	21,222 (27.4)	7,919 (22.2)	<0.001
3rd	21,178 (27.3)	10,162 (28.5)	<0.001
4th (highest)	16,625 (21.5)	11,322 (31.8)	<0.001
Missing	157 (0.2)	201 (0.6)	<0.001

[a] All Payers Claims Database

[b] The following races and ethnicities were included in the 'Other' category: Non-Hispanic American Indian or Alaskan Native, Non-Hispanic Asian, Non-Hispanic Native Hawaiian or Other Pacific Islander, and Non-Hispanic other (unspecified) race.

[c] Cases ≤10 have been masked to protect patient privacy

[d] Value has been masked to prevent obtaining counts in neighboring cells

© 2024 Sakai-Bizmark R et al. *JAMA Network Open*.
