# Supplementary material for: Short Interpregnancy Intervals Among Women Experiencing Homelessness in Colorado
Source: JAMA Netw Open. 2024 Jan 4;7(1):e2350242. doi: 10.1001/jamanetworkopen.2023.50242 (PMC10767616; doi:10.1001/jamanetworkopen.2023.50242)
Supplement: Supplement 2. — Data Sharing Statement [file jamanetwopen-e2350242-s002.pdf]

Data Sharing Statement

Sakai-Bizmark. Short Interpregnancy Intervals Among Women Experiencing Homelessness in Colorado. *JAMA Netw Open*. Published January 04, 2024.
doi:10.1001/jamanetworkopen.2023.50242

Data
Data available: No
